# Immunoregulatory Effects Triggered by Lactic Acid Bacteria Exopolysaccharides: New Insights into Molecular Interactions with Host Cells

**DOI:** 10.3390/microorganisms4030027

**Published:** 2016-08-15

**Authors:** Jonathan Laiño, Julio Villena, Paulraj Kanmani, Haruki Kitazawa

**Affiliations:** 1 Laboratory of Immunobiotechnology, Reference Centre for Lactobacilli (CERELA-CONICET), Tucuman CP 4000, Argentina; jonathan.laino@gmail.com; 2 Food and Feed Immunology Group, Laboratory of Animal Products Chemistry, Graduate School of Agricultural Science, Tohoku University, Sendai 981-8555, Japan; kanmanibiotech2007@gmail.com; 3 Livestock Immunology Unit, International Education and Research Center for Food and Agricultural Immunology (CFAI), Graduate School of Agricultural Science, Tohoku University, Sendai 981-8555, Japan

**Keywords:** lactic acid bacteria, immunobiotics, exopolysaccharides, PIE cells, TLR2, TLR4, RP105

## Abstract

Researchers have demonstrated that lactic acid bacteria (LAB) with immunomodulatory capabilities (immunobiotics) exert their beneficial effects through several molecules, including cell wall, peptidoglycan, and exopolysaccharides (EPS), that are able to interact with specific host cell receptors. EPS from LAB show a wide heterogeneity in its composition, meaning that biological properties depend on the strain and. therefore, only a part of the mechanism of action has been elucidated for these molecules. In this review, we summarize the current knowledge of the health-promoting actions of EPS from LAB with special focus on their immunoregulatory actions. In addition, we describe our studies using porcine intestinal epithelial cells (PIE cells) as a model to evaluate the molecular interactions of EPS from two immunobiotic LAB strains and the host cells. Our studies showed that EPS from immunobiotic LAB have anti-inflammatory capacities in PIE cells since they are able to reduce the production of inflammatory cytokines in cells challenged with the Toll-like receptor (TLR)-4-agonist lipopolysaccharide. The effects of EPS were dependent on TLR2, TLR4, and negative regulators of TLR signaling. We also reported that the radioprotective 105 (RP105)/MD1 complex, a member of the TLR family, is partially involved in the immunoregulatory effects of the EPS from LAB. Our work described, for the first time, that LAB and their EPS reduce inflammation in intestinal epithelial cells in a RP105/MD1-dependent manner. A continuing challenge for the future is to reveal more effector-receptor relationships in immunobiotic-host interactions that contribute to the beneficial effects of these bacteria on mucosal immune homeostasis. A detailed molecular understanding should lead to a more rational use of immunobiotics in general, and their EPS in particular, as efficient prevention and therapies for specific immune-related disorders in humans and animals.

## 1. Introduction

The term “exopolysaccharide” (EPS) is generally related to all forms of polysaccharides present outside of the microbial cell wall. EPS can be either weakly or strongly bound to the bacterial cell surface and they are distinguished into capsular and secreted forms. Several Gram-positive bacteria are able to produce EPS that participate in the protection of microbial cells against osmotic stress, desiccation, antibiotics, or toxic compounds, and phagocytosis [1]. Moreover, some EPS produced by bacteria are important biologic components in the interaction between microorganisms and the host through their capacity to mediate adhesion to surfaces, and cellular recognition.

Lactic acid bacteria (LAB) are able to synthesize EPS with a wide structural diversity. Bacterial EPS can be composed of one type of sugar monomer (homopolysaccharide) or consist of several types of monomers (heteropolysaccharide). Well-known examples of LAB homopolysaccharides, include dextrans and glucans produced by *Leuconostoc mesenteroides* and *Streptococcus mutans*, respectively. Heteropolysaccharides are synthesized by many LAB strains including *Streptococcus thermophilus*, *Lactococcus lactis*, and dairy *Lactobacillus* spp. [2]. Over the last decades, several researchers have demonstrated that LAB exert their beneficial effects on human and animal health through different molecules including cell wall, peptidoglycan, EPS, and secreted metabolites that are able to interact with specific host receptors [3,4,5]. In this regard, although EPS from LAB have found their most valuable application in the improvement of the rheology and texture of fermented milk products, it was reported that EPS produced by some strains of this group of microorganisms possess beneficial effects on health such as a decrease of blood cholesterol [6], immunostimulatory capacities [7,8], and antitumor activity [9]. Therefore, EPS from LAB have been received special attention as valuable compounds and received special attention in biotechnology and health applications. Several reviews on EPS produced by LAB have been published dealing mainly with the biosynthesis, chemical and structural characterization of EPS, as well as their biotechnological applications. To our criteria, the most relevant and detailed review covering these topics has been published recently by Torino et al. [10]. Some reviews have also addressed the health-promoting prebiotic effects of EPS from LAB strains [11], but their immunomodulatory activity and their molecular interaction with host cells have not been reviewed in detail.

This review aims to summarize the current knowledge of the beneficial immunomodulatory effects of EPS from LAB, especially those with the capacity to modulate inflammatory responses in the intestinal mucosa. We also discuss the role of pattern recognition receptors (PRRs), their signaling pathways, and their negative regulators in both the inflammatory intestinal injury and the beneficial effects of EPS from LAB.

## 2. Immune Health-Promoting Benefits of EPS from LAB

The immune system is able to differentiate between EPS produced by pathogens and commensal bacteria [12]. In the last two decades, EPS produced by bacterial pathogens have received considerable scientific attention, mainly due to their contribution to biofilm formation, and because their potential as virulence factors. Recently, EPS from commensal/beneficial bacteria have received attention because of their capacity to mediate communication with their surrounding environment including host’s cells, and their contribution to health maintenance. In fact, some studies reported that EPS produced by commensal and probiotic bacteria are able to modulate systemic and mucosal immune responses, and in turn to provide direct health-promoting benefits. In this regard, the biosynthesis of EPS has been described in several species of LAB strains including *Bifidobacterium*, *Lactobacillus*, *Lactococcus*, and *Leuconostoc*, and it seems that all of these molecules were found to play a relevant role in their immunoregulatory capabilities (Table 1).

EPS from LAB show a wide heterogeneity in its composition, meaning that their biological properties depend on the strain and, therefore, only a part of their mechanisms of action has been elucidated. Some EPS of high molecular weight are able to induce the activation of immune cells including dendritic cells (DCs), macrophages, and splenocytes; and to induce the production of specific cytokines. In general, it was observed that negative charged EPS and/or small size molecules would act as stimulators of immune cells, while neutral and big size EPS would have a suppressor effect [13].

Some in vivo studies on animal models have clearly demonstrated that EPS produced by commensal bacteria or LAB are able to increase protection against pathogens. Recently, it was demonstrated that polysaccharides from *Bacteroides fragilis* protect animals from experimental colitis induced by *Helicobacter hepaticus* by suppressing the production of IL-17, and stimulating the regulatory T cells to produce IL-10 [14]. By using an infection model with *Citrobacter rodentium*, a murine attaching and effacing (A/E) pathogen related to human diarrheagenic A/E *Escherichia coli*, Fanning et al. [15] found that EPS from *B. breve* UCC2003 is able to diminish pathogen colonization, and that this effect was related to an improvement of IL-12 and increases in antibody-producing cells. Another example of immune system regulation by EPS of LAB was reported by Nikolic et al. [16], who investigated the probiotic effects of the EPS producing strain *L. paraplantarum* BGCG11. The work demonstrated that the presence or absence of EPS in the BGCG11 strain significantly altered the immune response of human peripheral blood mononuclear cells (PBMC) since the authors showed that the EPS-producing *L. paraplantarum* BGCG11 strain induced an anti-inflammatory profile, while the non-EPS-producing derivative induced a pro-inflammatory response. Similarly, it was reported that bifidobacterial EPS-purified fractions stimulated the proliferation of PBMC and that strains producing neutral high molecular weight EPS induced the production of IL-10 [17]. In addition, the high molecular weight EPS from *L. rhamnosus* RW-9595M induced low levels of pro-inflammatory mediators in macrophages and lymphocytes, while increased the production of the immunoregulatory cytokine IL-10 [18]. The anti-inflammatory capabilities of some EPS from LAB had been used to induce the desensitization of macrophages [19]. It was demonstrated that pre-treatment of macrophages with EPS reduced TNF-α production after re-stimulation with LPS. This phenomenon called “cross-tolerance” was also observed after re-stimulation with other stimulus [19].

On the other hand, some reports demonstrated immunostimulatory effects for EPS from LAB. The EPS from a *L. paracasei* strain has the ability to induce the production of IL-6, IL-1β, and TNF-α in murine RAW macrophages [20]. Additionally, it was observed that EPS was able to promote phagocytosis by RAW cells. In addition, EPS from *L. plantarum* NTU102 upregulated the mRNA level of iNOS in RAW macrophages through the activation of NF-κB, and induced cytokine production (TNF-α, IL-1β, and IL-6) [20]. It was reported that heteropolysaccharides with phosphate groups (negative charge) are good inducers of immune responses, by stimulating the synthesis of TNF-α and IL-1α in spleen macrophages in vitro [21]. In addition, remarkable differences have been found in the immunomodulatory activities of acidic (APS) and neutral (NPS) EPS from LAB strains [9,22,23]. The APS (phosphopolysaccharide) of *L. bulgaricus* OLL1073-R1 improved macrophages function in vivo and in vitro, while the NPS only showed a weak effect in vitro. APS was found to be a potent stimulator of macrophages by inducing the production of several cytokines. This effect was significantly reduced after removal of phosphorus from APS.

As described above, a number of EPS from LAB have been shown to exhibit immunomodulatory activity with professional immune cells, but only limited studies have been reported of their interaction with intestinal epithelial cells (IECs). Those studies are important considering that IECs are able to functionally modulate mucosal immune responses and are one of the first cells that take contact with immunobiotics besides mucosal immune cells. Most studies evaluating the interaction of EPS from LAB and IECs focused in the capacity of EPS-producing LAB to adhere to cells, however the impact of those molecules in the immunobiology of IECs has not been studied in detail. Patten et al. [24], evaluated the effect of neutral EPS from *L. helveticus* ssp. *rosyjski* and *L. acidophilus* in the HT29-19A intestinal epithelial cell line and demonstrated that those molecules were capable to induce IL-8 production, which suggested a beneficial effect of EPS on gut homeostasis in vivo.

## 3. Porcine Intestinal Epithelial Cells and Inflammation

In the last decades, there was an increasing interest in the porcine immune system because of the great importance of swine as livestock and for its potential use as a model for the human immune system [25,26]. Pigs share similarities in anatomy, physiology, and genetics with humans and, therefore, they are considered an important biomedical model for human immune responses and diseases [27,28,29,30]. Particularly, the porcine gastrointestinal tract has many structural aspects that are more similar to those of the human system than the rodent system [30]. Therefore, our research group has focused on the porcine gut immune system as a human model and has studied the immune responses in intestinal epithelial and immune cells via PRRs.

IECs are a central component of the gastrointestinal immune system. Several works clearly demonstrated that microbial recognition by IECs is an integral aspect of first-line host responses. In order to study the contribution of IECs to the induction of immune responses against gastrointestinal pathogens or beneficial immunoregulatory microorganisms, we established a clonal porcine intestinal epitheliocyte cell line (PIE cells) [31]. Evaluation of PRRs expression in PIE cells revealed that TLR4 was strongly expressed, and our studies confirmed that these cells undergo inflammatory responses regarding cytokine and chemokine expression in response to TLR4 activation by LPS [31] or pathogenic Gram-negative bacteria [32].

**Figure 1:**
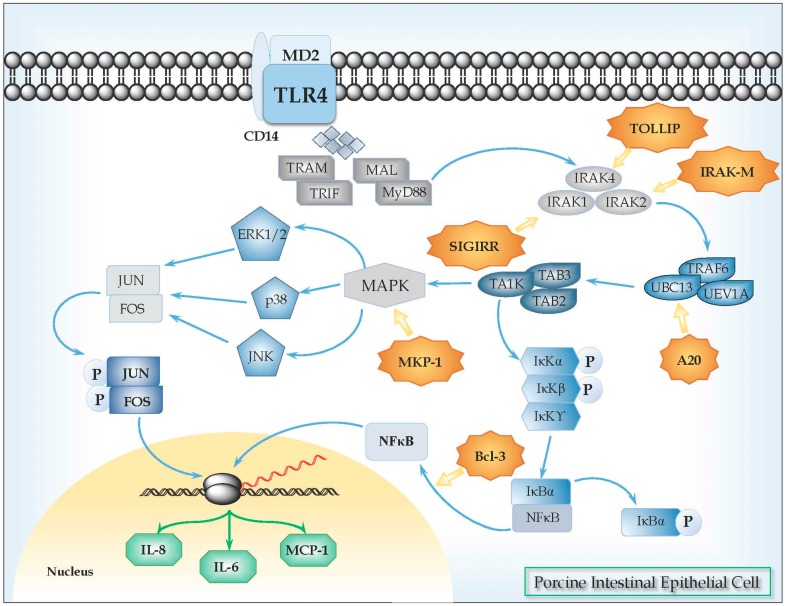
Toll-like receptor 4 (TLR4) signaling pathway in porcine intestinal epithelial (PIE) cells. Upon recognition of its cognate ligand, TLR4 dimerizes and initiates a signaling cascade that leads to the activation of a pro-inflammatory response. Ligand binding can induce two signaling pathways, the myeloid differentiation primary response gene 88 (MyD88)-dependent and MyD88-independent pathways, which induce the production of pro-inflammatory cytokines and type I IFNs. In MyD88-dependent signaling, upon ligand recognition, MyD88 is recruited to, and associates, with the cytoplasmic domain of the TLRs. Then IL-1R-associated kinase 4 (IRAK-4) and IRAK-1 are recruited and activated by phosphorylation. Activated IRAK-4 phosphorylates IRAK-1, which subsequently associates with tumor necrosis factor receptor (TNFR)-associated factor 6 (TRAF6). TRAF6 activates transforming growth factor (TGF)-activating kinase 1 (TAK1). TAK1 phosphorylates IKK-b and mitogen-activated protein kinase (MAPK) kinase 6 (MKK6), leading to degradation of I-κB and thereby leading to the nuclear translocation of NF-κB, which results in the induction of genes involved in inflammatory responses. Activation of the MyD88-dependent pathway also results in the activation of MAPKs such as p38 and JNK, which leads to the activation of AP-1 (Jun/Fos). Various negative regulatory mechanisms have evolved to attenuate TLR signaling and maintain the immune balance, including the activity of the TLR-negative regulators TOLLIP, IRAK-M, SIGIRR, MKP-1, A20, and Bcl3.

We have also characterized the TLR4 signaling pathway in PIE cells and showed that it is similar to those described for human and mice IECs [33] (Figure 1). Upon activation by their ligands, TLR4 dimerizes and initiates a signaling cascade that leads to the activation of a pro-inflammatory response. Ligand binding mediates signaling by two possible pathways, which includes the myeloid differentiation primary response gene 88 (MyD88)-dependent and MyD88-independent pathways. The MyD88-dependent signaling leads to IKK-β phosphorylation, leading to degradation of I-κB, and nuclear translocation of NF-κB [33]. In addition, the MyD88-dependent pathway also results in the activation of MAPKs, such as p38 and JNK, which leads to the activation of AP-1 [33]. Both, NF-κB and MAPK pathways are activated in PIE cells after activation of TLR-4 [34].

Regulatory mechanisms have evolved to modulate TLRs signaling and maintain the immune balance, inducing protection against inflammatory damage [35]. In this regard, we have characterized some negative regulators of TLRs signaling pathway in PIE cells (Figure 1, Table 2). In order to evaluate the expression of these TLRs negative regulators, cDNAs corresponding to these porcine proteins were cloned [36]. Nucleic acid sequences and the deduced amino acid of porcine SIGIRR, Tollip, A20, Bcl-3, MKP-1, and a partial porcine IRAK-M ORF were compared to those from humans and mice. We also evaluated the expression of SIGIRR, Tollip, A20, Bcl-3, MKP-1, and IRAK-M mRNAs in PIE cells and found that all of them are expressed and functional in this cell line [34].

## 4. Anti-Inflammatory Effects of EPS from LAB in PIE Cells

Considering that PIE cells are able to express functional TLR4 and negative regulators of TLRs signaling, we used this porcine cell line to perform a selection LAB with the ability to beneficially modulate the immune response triggered by TLR4 activation, and to deepen the knowledge of the molecular mechanisms involved in their immunoregulatory activity [34,37,38]. By using LPS or heat-stable enterotoxigenic *Escherichia coli* pathogen-associated molecular patterns and this porcine in vitro model system we selected immunomodulatory LAB strains including *L. jensenii* TL2937, *B. breve* MCC-117, and *B. longum* BB536 that are able to improve protection against TLR4-mediated inflammatory damage in PIE cells. Our research work demonstrated that treatment of PIE cells with immunobiotic bacteria before the challenge with TLR4 agonists enhanced the expression of some negative regulators of TLRs, modulated NF-κB and MAPK pathways, and suppressed pro-inflammatory cytokine and chemokine production [34,38].

More recently, we focused our studies in two bacteria: *Lactobacillus plantarum* N14 [39] and *Lactobacillus delbrueckii* TUA4408L [33], which are lactobacilli strains able to produce immunomodulatory EPS. *L. plantarum* N14 is an immunobiotic strain with the capacity to improve Th1 responses exerting anti-allergic and immunostimulatory effects [40,41]. On the other hand, *L. delbrueckii* TUA4408L, which was originally isolated from a Japanese traditional fermented pickle (sunki), has shown immunomodulatory effects in pigs [42].

*L. plantarum* N14 and *L. delbrueckii* TUA4408L are able to downregulate pro-inflammatory cytokines expressions (IL-6, IL-8, and MCP-1) in PIE cells after TLR4 activation. Similarly to other immunobiotic bacteria [33], their anti-inflammatory activities were related to the capacity of N14 or TUA4408L strains to modulate the activation of NF-κB and MAPK pathways [33,39]. Moreover, our studies demonstrated that the immunoregulatory activities of these two lactobacilli were related to the induction of negative regulators of the TLR signaling [33,39]. Of interest, we found that EPS are key molecules for the anti-inflammatory effects of both *L. plantarum* N14 and *L. delbrueckii* TUA4408L.

EPS produced by N14 and TUA4408L strains can be fractionated into two types of EPS that had a different sugar composition. NPS and APS from N14 and TUA4408L can be fractionated by anion exchange chromatography [33,39,41]. Our research showed that NPS and APS from *L. plantarum* N14 and *L. delbrueckii* TUA4408L are able to activate PRRs expressed in PIE cells, upregulate negative regulators of TLRs signaling, and reduce the production of inflammatory mediators after activation of TLR4 [33,39]. We found that three PRRs are involved in the activities of those NPS and APS: TLR2, TLR4, and RP105.

## 5. Role of TLR2 in the Immunomodulatory Effects of NPS from Lactobacilli

Host–immunobiotic interactions involve a complex crosstalk between various microbial molecules and host receptors. TLR2 is among the receptors that have been widely studied to explain the immunomodulatory effects of probiotic bacteria. In vitro studies showed that *B. pseudocatenulatum* CECT7765 inhibits the production of inflammatory cytokines (TNF-α and IL-6) induced by immunostimulatory lactobacilli in blood immune cells via interaction with TLR2 [73]. Moreover, anti-inflammatory *B. longum* Q46 induces much lower levels of IL-10 and higher IL-12 levels in bone marrow-derived DCs from TLR2 knockout mice compared with wild-type DCs [73]. Finamore et al. [74] demonstrated that *L. amylovorus* DSM 16698T modulates TLR4 activation in Caco-2 cells through modulation of the negative regulators Tollip and IRAK-M and, that this effect was dependent on TLR2 signaling. More recently, in vivo studies performed by Thakur et al. [75] in a mouse model of experimental colitis, demonstrated that both live and heat-killed *L. casei* Lbs2 are able to activate DCs thought TLR2 and polarize naive T cells to Treg cells increasing the frequency of FoxP3^+^ T cells. Immunobiotic treatment reduced the levels of TNF-α, IL-12, and IL-17A, while increased IL-10 and TGF-β in colonic tissues. Those changes induced by the Lbs2 strain significantly ameliorated the disease manifestations of murine colitis. By using a model of intestinal inflammation in mice with cirrhosis, Moratalla et al. [35] showed that administration of *B. pseudocatenulatum* CECT7765 significantly reduced intestinal permeability, decreased the burden of bacterial antigens in the liver and improved liver function. Those effects were related to the capacity of the CECT7765 strain to reduce the expression of chemokine receptors CXCR6, CXCR3, CCR9, and CCR6 in intestinal lymphocytes and, induce higher and lower levels of IL-10 and TNF-α, respectively. Similarly, to the previous described works, authors demonstrated the immunoregulatory effect of *B. pseudocatenulatum* CECT7765 was dependent of TLR2 activation.

**Figure 2:**
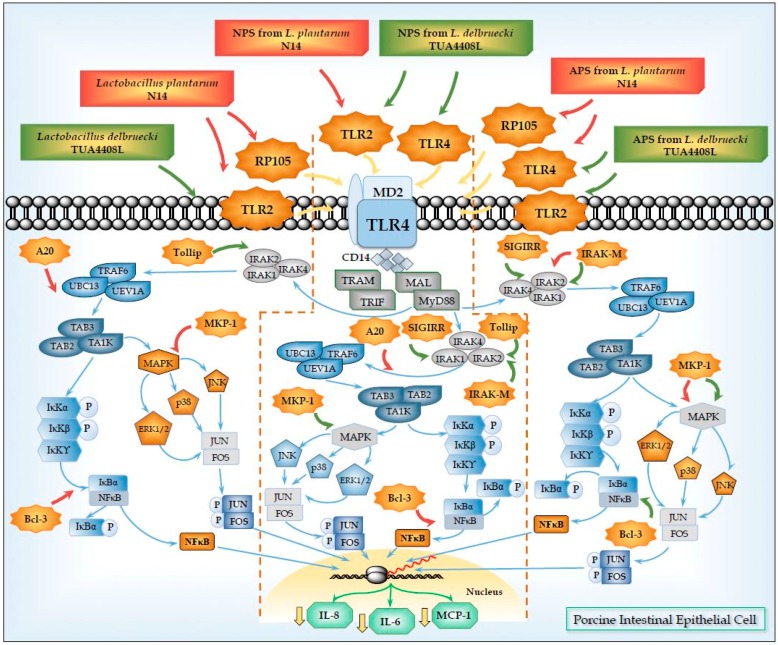
Modulation of Toll-like receptor 4 (TLR4) signaling pathway in porcine intestinal epithelial (PIE) cells by acidic (APS) and neutral (NPS) exopolysaccharides produced by *Lactobacillus plantarum* N14 and *Lactobacillus delbrueckii* TUA4408L. Treatment of PIE cells with *L. plantarum* N14, *L. delbrueckii* TUA4408L or their APS and NPS induce the expression of TLR negative regulators TOLLIP, IRAK-M, SIGIRR, MKP-1, A20, and/or Bcl3 via TLR2, TLR4, and/or RP105. Treated-PIE cells showed a reduced inflammatory response in terms of chemokines and cytokines production after the subsequent challenge of with TLR4 agonists.

Those studies clearly showed a key role for TLR2 in the anti-inflammatory effect of immunobiotic bacteria. In agreement, our studies in PIE cells and porcine immune cells also revealed that TLR2 plays an important role in the immunoregulatory activity of immunobiotic bacteria [34,38,76,77]. Our studies of the anti-inflammatory activity of *L. jensenii* TL2937, *B. breve* MCC-117, and *B. longum* BB536 in PIE cells showed that TLR2 is necessary for the immunoregulatory capacities of those bacteria. In fact, our work demonstrated that those strains are able to increase A20 expression and reduced the production of pro-inflammatory cytokines in PIE cells after TLR4 activation in a TLR2-dependent manner [34,38]. Similarly, we demonstrated that TLR2 has a significant role in the immunoregulatory effect of *L. plantarum* N14 [39], *L. delbrueckii* TUA4408L [33], and their NPS in PIE cells (Figure 2).

We demonstrated that NPS from *L. plantarum* N14 or *L. delbrueckii* TUA4408L were able to modulate TLR4-induced expression of pro-inflammatory cytokines and chemokines in PIE cells in a TLR2-dependent manner [33,39]. The human HEK293 cell line was transfected with plasmids encoding porcine TLR2 in order to obtain HEK^TLR2^ cells. The NF-κB reporter assay and the induction of intracellular Ca^2+^ fluxes performed in these cells allowed us to demonstrate the ability of NPS from N14 strain to stimulate TLR2. Moreover, we used RNA interference technology to knockdown TLR2 in PIE cells and showed that the capacity of NPS to improve the expression of TGF-β in PIE cells was reduced in PIE^TLR2−/−^ cells [39]. Similarly, we observed that blocking anti-TLR2 antibodies abolished the ability of NPS from *L. delbrueckii* TUA4408L to reduce IL-8 in PIE cells after TLR4 activation [33]. Our results also indicated that NPS would be partially responsible of the immunomodulatory effects of *L. plantarum* N14 and *L. delbrueckii* TUA4408L through their capacity to activate TLR2 since we observed that both bacteria are able to reduce the expression of pro-inflammatory mediators triggered by TLR4 in PIE cells in a TLR2-dependent manner cells (Figure 2).

In support of our findings, some works described the recognition of bacterial polysaccharides by TLR2 [78,79]. *Thermus aquaticus*, a Gram-negative bacteria able to from a biofilm, produces an extracellular polysaccharide that is recognized by TLR2. This extracellular polysaccharide/TLR2 interaction was demonstrated by the production of IL-6 in peritoneal macrophages from wild-type mice but not from TLR2^−/−^ mice [78]. It was also reported that recognition of *Streptococcus suis* capsular polysaccharide depends on TLR2 [79]. Authors used human monocytes and showed that stimulation of these cells with *S. suis* capsular polysaccharide induced the production of MCP-1, IL-8, IL-6 and, IL-1. Moreover, the release of these pro-inflammatory mediators triggered by the polysaccharide was significantly reduced by antibody-mediated blocking of TLR2.

## 6. Role of TLR4 and RP105 in the Immunomodulatory Effects of APS from Lactobacilli

In addition to TLR2, we described, for the first time, that the radioprotective 105 (RP105)/MD1 receptor complex could mediate the immunomodulatory effect of EPS from immunobiotic bacteria. In fact, our research work with the APS from *L. plantarum* N14 demonstrated that RP105/MD1 is partially involved in the upregulation of the expression of IRAK-M and MKP-1; and the reduction of IL-6, IL-8, and MCP-1 expression in APS-treated PIE cells after the activation of TLR4 [39] (Figure 2).

RP105 (CD180) was first described in B cells from mice [80]. However, it was demonstrated later that RP105 is not B cell-specific since this receptor is expressed in myeloid cells, including macrophages and DCs, in a similar way to TLR4 [81]. Molecular analysis of human and mouse RP105 revealed a type I transmembrane protein that is structurally similar to TLRs. RP105 contains extracellular leucine-rich repeat domains and the pattern of juxtamembrane cysteine residues conserved among the TLRs and therefore, this receptor is considered a member of the TLR family of proteins. However, in contrast to TLRs, RP105 lacks a TIR domain [82]. Functional studies revealed that RP105 shares similarities to TLR4. Signaling through RP105 is dependent on a secreted, extracellularly associated accessory protein, MD1 [83]. Of interest, it was reported that RP105/MD1 complex inhibits LPS–TLR4/MD2 signaling complex formation in HEK cells co-expressing both receptors and, for this reason RP105 is considered also a negative regulator of TLR4 signaling [84,85]. In line with this observation, it was demonstrated RP105 inhibit activation of TLR4 induced by LPS in mouse DCs and macrophages, reducing the production of cytokines and that the challenge with LPS significantly increased the systemic production of TNF-α in RP105^−/−^ mice [84,85]. More recently, Wezel et al. [86] showed that vein graft disease was aggravated in RP105^−/−^ mice and that this effect was the result of an increased local inflammatory response. In fact, RP105 deficiency induced macrophages, smooth muscle cells, and mast cells to secrete higher levels of CCL2 increasing inflammatory damage.

There is emerging evidence for involvement of RP105 in the host response to infection, including acute influenza and pathogenic mycobacteria infection; however, the functional relevance of this receptor in protection or contribution to pathogenesis during infectious diseases remains to be elucidated [87]. In addition, contributions of RP105/MD2 complex to inflammatory disorders not directly connected to infectious processes have been described for obesity, atherosclerosis, and arthritis [88]. However, the role of RP105 in the immunomodulatory effect of beneficial bacteria has not been described before.

We cloned porcine RP105 and porcine MD1 from ileal Peyer’s patches and determined the full-length cDNA sequences of both genes [88]. The nucleotide and aminoacid sequences of porcine RP105 and MD1 were more similar to those of human than those of mouse, supporting the idea that swine is a better model than the mouse for human immune system extrapolations [88]. Subsequently, we constructed HEK^RP105/MD1^ cells and demonstrated that the RP105/MD1 complex is involved in the recognition of phosphopolysaccharides produced by an immunobiotic *Lactococcus lactis* ssp. *cremoris* strain. Interaction of phosphopolysaccharides with the RP105/MD1 complex resulted in the activation of NF-κB through the phosphatidylinositol 3-kinase (PI3K) and Bruton’s tyrosine kinase (Btk) signaling pathway [88].

In addition, the role of RP105/MD1 in the immunobiology of IECs was not described before. We observed that this receptor complex is expressed and is functional in PIE cells [39]. Moreover, our results demonstrated that RP105/MD1 is necessary for the production of pro-inflammatory cytokines by PIE cells in response to LPS or Gram negative bacteria [39]. Of interest, as we mentioned before we were also able to prove that RP105/MD1 has an important role in the anti-inflammatory effect of APS from *L. plantarum* N14 in PIE cells after the activation of TLR4. In line with this finding, APS from the N14 strain significantly increased the mobilization of Ca^2+^ in HEK^RP105/MD1^ cells Moreover, when we used RNA interference technology to knockdown RP105 gene expression in PIE cells we found that the capacity of APS to modulate IL-8 and IL-6 production was abolished in PIE^RP105/MD1−/−^ cells [39].

On the other hand, our studies also showed that immunoregulatory effects of APS from *L. plantarum* N14, and APS and NPS from *L. delbrueckii* TUA4408L were partially dependent on TLR4 [33,39].

The immunoregulatory/anti-inflammatory effects of oligosaccharides through TLR4 activation have been reported before [89,90]. Fructooligosaccharides and inulin effects were evaluated in splenocytes from TLR4^−/−^ and wild-type mice [89]. Both polysaccharides induced the production of cytokines including TNF-α, IL-6, and, IL-10 by mouse splenocytes, but inhibited LPS-induced IFN-γ and IL-17 release. Interestingly, splenocytes from TLR4^−/−^ mice showed a markedly depressed response, indicating that both fructooligosaccharides and inulin act as TLR4 ligands. In addition, by using an LPS-induced model of nonlethal endotoxemia in mice, Kovacs-Nolan et al. [90], demonstrated that the non-digestible disaccharide β-1,4-mannobiose has anti-inflammatory effects. Authors reported that β-1,4-mannobiose is able to act as a TLR4 ligand. Moreover, oligosaccharides are also able to modulate innate immunity in IECs. It was documented that oligosaccharides reduce the expression of the pro-inflammatory cytokines IL-12p35, IL-8, and TNF-α in Caco-2 cells, indicating that oligosaccharides has the ability to modulate IECs immune response. This effect was achieved through inhibition of NF-κB and, activation of PPARγ [91]. It was also reported recently that inulin, goat’s milk oligosaccharides or galactooligosaccharides modulated the production of pro-inflammatory mediators in IECs lines. Interestingly, that work demonstrated, for the first time, that these prebiotic oligosaccharides act as TLR4 ligands in IECs and that the immunoregulatory effects were dependent on TLR4, Myd88, and NF-κB [92].

In line with those previous findings, we demonstrated that both APS and NPS isolated from *L. delbrueckii* TUA4408L, and APS from *L. plantarum* N14 were able to downregulate the expression of the pro-inflammatory cytokines IL-6, IL-8, and MCP-1 in PIE cells after TLR4 activation. Interestingly, the three treatments increased the expression of IRAK-M and, MKP-1, and reduced the activation of NF-κB, and MAPK pathways [33,39]. Our experiments using blocking antibodies, knockdown PIE cells, and calcium mobilization, showed that the immunoregulatory capacities of those EPS were dependent on TLR4 signaling that occurred before the second challenge with TLR4 agonists (Figure 2). Then, we have described for the first time that EPS from LAB are able to reduce inflammation in porcine IECs in a TLR4-dependend manner through the regulation in the expression of negative regulators of the TLR signaling. Moreover, our results provide evidence for a key role for IRAK-M and MKP-1 in the anti- inflammatory effect of EPS from lactobacilli.

## 7. Conclusions

Our studies with EPS from *L. plantarum* N14 and *L. delbrueckii* TUA4408L showed that negative regulators of TLRs signaling are key players in the activity of immunoregulatory EPS from lactobacilli. The combination of TLRs negative regulators that was upregulated in PIE cells was specific for each EPS, which would explain the different capacity of APS and NPS isolated from *L. delbrueckii* TUA4408L or *L. plantarum* N14 to modulate the NF-kB and MAPK pathways. This differential effect could certainly be due to the molecular complexity of the different EPS that may contact and activate the various PRRs expressed in PIE cells (TLR2, TLR4, or RP105), resulting in changes in PIE cells that are characteristic for each EPS.

Our work also provides new information regarding the mechanism involved in the anti-inflammatory effect of immunobiotics by demonstrating that different immunoregulatory EPS from lactobacilli use a common mechanism to induce tolerance in PIE cells. Immunoregulatory EPS from lactobacilli interact with RP105 and/or TLR4, upregulate the expression of IRAK-M and MKP-1 in PIE cells, and beneficially modulate the subsequent TLR4 activation by reducing the activation of MAPK and NF-kB pathways and the production of pro-inflammatory cytokines. Then, our results demonstrated that the combination of RP105 and TLR4 activation together with IRAK-M and MKP-1 induction could be used as biomarkers to screen and select potential immunoregulatory EPS from lactobacilli strains.

## Figures and Tables

**Table 1:** Immunomodulatory effects of EPS from lactic acid bacteria.

Immunological Effects	Host	Strain	References
Induction of cytokine production by macrophages, especially TNF-α, IL-6, and IL-12. Desensitization of macrophages. Decrease of TNF-α production after re-stimulation with EPS.	Murine peritoneal macrophages	*Lactobacillus rhamnosus* KL37	[43]
Modulation of immune cell recruitment and cytokine production. Reduction of *Citrobacter rodentium* colonization.	Mice	*Bifidobacterium breve* UCC2003	[15]
Down-regulation of inflammatory response.	Human peripheral blood mononuclear cells	*Lactobacillus paraplantarum* BGCG11	[16]
Immunostimulatory effect on macrophages and lymphocytes. Increase of pro-inflammatory cytokines expression, mainly IL-8.	HT29-19A cell line	*Lactobacillus helveticus* sp. *Rosyjski* *Lactobacillus acidophilus* sp. 5e2	[24]
Stimulation of immune response. Mitogenic activity. Cytotoxicity. Induction of INF-γ, and IL-1α synthesis on spleen macrophages.	B lymphocytes and murine macrophages	*Lactococcus lactis* subsp. *cremoris* KVS20	[21,44,45,46,47]
Increase of macrophage phagocytic activity. Increase of murine splenocytes mitogenic activity. Enhancement of macrophages cytotoxicity against tumour cells. Induction of cytokine production in macrophages.	Murine lymphocytes and murine macrophages including cell line J774.1	*Lactobacillus bulgaricus* OLL1073-R1	[9,23,48]
Reduction of immune cells reaction against LPS. Decrease in the production of TNF-α, IL-12, IL-10, and IL-6.	Murine spleen cells and murine RAW macrophages	*Lactobacillus casei* Shirota	[49]
Reduction of TNF-α, IL-6, and IL-12. Induction of high levels of IL-10.	Murine macrophages and splenic lymphocytes	*Lactobacillus rhamnosus* RW-9595M *Lactobacillus rhamnosus* ATCC9595	[18]
Induction of tolerogenic dendritic cells. Improvement in the production of immunosuppressor cytokines. Expansion of regulatory Foxp3^+^CD25^hi^ Treg cells. Control of Th17 cells differentiation.	Mice	*Bifidobacterium animalis* subsp. *lactis* IPLA-R1	[13]
Induction of IL-6, IL-1β, and TNF-α. Promotion of phagocytosis, and increase of NO.	Murine RAW macrophages	*Lactobacillus paracasei* NTU101 *Lactobacillus plantarum* NTU102	[20]
Stimulation of IgA production.	Mice	*Leuconostoc mesenteroides* NTM048	[50]

**Table 2:** Inhibitors of Toll-like receptor signaling.

Regulator	Name	Described Effects	References
SIGIRR	Single immunoglobulin interleukin-1 related receptor	SIGIRR acts as a negative regulator of IL-1 and TLR signaling. High expression of SIGIRR in epithelial cells indicates that SIGIRR may serve mainly to decrease the immune response in cells that are continually exposed to microorganisms, such as colon and lung epithelial cells. SIGIRR is an important modulator of intestinal epithelial homeostasis and a key regulator of mucosal immunity, maintaining microbial tolerance of the intestinal epithelial layer.	[51,52,53,54,55]
Tollip	Toll interacting protein	Tollip was associated with TLR2 and TLR4 and play an inhibitory role in TLR-mediated cell activation. The primary role of Tollip-mediated pathway may be to maintain immune cells in a quiescent state in the absence of infection and facilitate the termination of TLR-induced cell signaling during inflammation and infection.	[56]
A20 (TNFAIP3)	Tumor necrosis factor alpha-induced protein-3	A20 is a zinc finger protein that functions via its two ubiquitin-editing activities. These two activities cooperatively down-regulate TRAF6 and terminate NF-kB signaling. A20 plays an essential role in the response to TNF-α and microbial products such as LPS. Inhibitor of NF-κB signaling induced by TNF-α, IL-1, CD40, PRRs, and T cell and B cell antigen receptor activation.	[57,58,59,60,61,62,63]
Bcl-3	B-cell lymphoma-3	Bcl-3 functions as an inhibitor of NF-κB activity by stabilizing repressive NF-κB homodimers in a DNA-bound state and preventing the binding of transcriptionally active dimers. Repressive complexes through the induction of Bcl-3 expression has been proposed to function during the processes of LPS tolerance. Bcl-3 has been reported to be involved in restricting inflammation by both suppressing IL-23 and inducing IL-10.	[64,65,66,67]
MKP-1	Mitogen-activated protein kinase phosphatase-1	MKP-1 plays a role in the inhibition of pro-inflammatory mRNA expression by inactivating MAPK. MKP-1 desensitizes cells to TLR ligands by inactivating p38 signaling pathway in enterocytes. MKP-1 desactivates MAPK (ERK, JNK, p38) by dephosphorilation.	[68,69,70]
IRAK-M	Interleukin-1 receptor-associated kinase M	IRAK-M is thought to bind MyD88/IRAK-4 and inhibit IRAK-4 phosphorylation of IRAK-1. This prevents formation of TRAF6/IRAK-1 complexes, which initiate IκB kinase and MAPK signaling pathways. IRAK-M-dependent pathway only induces expression of genes that are not regulated at the post-transcriptional levels (including inhibitory molecules SOCS-1, SHIP-1, A20 and IkBa), exerting an overall inhibitory effect on inflammatory response. The interaction of IRAK-M with IRAK-2 also suppresses inflammation, by suppressing cytokine and chemokine production.	[71,72]
